# Non-Ebola Filoviruses: Potential Threats to Global Health Security

**DOI:** 10.3390/v16081179

**Published:** 2024-07-23

**Authors:** Yannick Munyeku-Bazitama, Francois Edidi-Atani, Ayato Takada

**Affiliations:** 1 Division of Global Epidemiology, International Institute for Zoonosis Control, Hokkaido University, Sapporo 001-0020, Japan; ymunyeku@gmail.com (Y.M.-B.); edidi@czc.hokudai.ac.jp (F.E.-A.); 2 Institut National de Recherche Biomédicale, Kinshasa P.O. Box 1197, Democratic Republic of the Congo; 3 Département de Biologie Médicale, Faculté de Médecine, Université de Kinshasa, Kinshasa P.O. Box 123, Democratic Republic of the Congo; 4 International Collaboration Unit, International Institute for Zoonosis Control, Hokkaido University, Sapporo 001-0020, Japan; 5 One Health Research Center, Hokkaido University, Sapporo 001-0020, Japan; 6 Department of Disease Control, School of Veterinary Medicine, University of Zambia, Lusaka 10101, Zambia

**Keywords:** Filovirus, non-Ebola filovirus, Sudan virus, Marburg virus, Ravn virus, Reston virus, Taï Forest virus, Bundibugyo virus, Bombali virus, Lloviu virus, Mengla virus, Dehong virus

## Abstract

Filoviruses are negative-sense single-stranded RNA viruses often associated with severe and highly lethal hemorrhagic fever in humans and nonhuman primates, with case fatality rates as high as 90%. Of the known filoviruses, Ebola virus (EBOV), the prototype of the genus *Orthoebolavirus*, has been a major public health concern as it frequently causes outbreaks and was associated with an unprecedented outbreak in several Western African countries in 2013–2016, affecting 28,610 people, 11,308 of whom died. Thereafter, filovirus research mostly focused on EBOV, paying less attention to other equally deadly orthoebolaviruses (Sudan, Bundibugyo, and Taï Forest viruses) and orthomarburgviruses (Marburg and Ravn viruses). Some of these filoviruses have emerged in nonendemic areas, as exemplified by four Marburg disease outbreaks recorded in Guinea, Ghana, Tanzania, and Equatorial Guinea between 2021 and 2023. Similarly, the Sudan virus has reemerged in Uganda 10 years after the last recorded outbreak. Moreover, several novel bat-derived filoviruses have been discovered in the last 15 years (Lloviu virus, Bombali virus, Měnglà virus, and Dehong virus), most of which are poorly characterized but may display a wide host range. These novel viruses have the potential to cause outbreaks in humans. Several gaps are yet to be addressed regarding known and emerging filoviruses. These gaps include the virus ecology and pathogenicity, mechanisms of zoonotic transmission, host range and susceptibility, and the development of specific medical countermeasures. In this review, we summarize the current knowledge on non-Ebola filoviruses (Bombali virus, Bundibugyo virus, Reston virus, Sudan virus, Tai Forest virus, Marburg virus, Ravn virus, Lloviu virus, Měnglà virus, and Dehong virus) and suggest some strategies to accelerate specific countermeasure development.

## 1. Introduction

Filoviruses belong to the *Filoviridae* family, which includes enveloped negative-sense single-stranded RNA viruses infecting fish, mammals, and reptiles [1,2]. Their genome sizes are about 13.1–20.9 kb and consist of a linear, non-segmented RNA encoding at least five essential proteins: a nucleoprotein (NP), a polymerase cofactor (VP35), an envelope glycoprotein (GP), a transcriptional activator (VP30), and a large protein (L) that contains an RNA-dependent RNA polymerase (RdRP) domain [1,2]. Additional proteins, such as a matrix protein (VP40) and an interferon-antagonist (VP24), are encoded depending on the genus [1,2]. Filoviruses are assigned into 9 genera associated with mammalian hosts (*Orthoebolavirus*, *Orthomarburgvirus*, *Cuevavirus*, and *Dianlovirus*), fish hosts (*Oblavirus*, *Striavirus*, *Thamnovirus*, and *Loebevirus*), or reptilian hosts (*Tapjovirus*) [1,2]. Most orthoebolaviruses and orthomarburgviruses have been associated with severe hemorrhagic fever outbreaks in humans and nonhuman primates (NHPs). Of these, the Ebola virus (EBOV), representing the genus *Orthoebolavirus zairense*, caused an unprecedented Ebola disease outbreak in 2013–2016 in West Africa (Guinea, Liberia, and Sierra Leone) with 28,610 cases, including 11,308 deaths (case fatality rate (CFR) = 39%) [3]. This outbreak popularized global health security because of its regional (Mali, Nigeria, and Senegal) and international (Spain, the United Kingdom, and the United States) spread and resulted in substantial funding that accelerated basic and translational research on EBOV [3,4]. EBOV research was therefore prioritized, leaving behind other orthoebolaviruses (Sudan virus (SUDV), Bundibugyo virus (BDBV), and Taï Forest virus (TAFV)), and orthomarburgviruses (Marburg virus (MARV) and Ravn virus (RAVV)) causing serious outbreaks. Indeed, MARV recently emerged in new areas of Africa between 2021 and 2023 [5], as did SUDV in Uganda in 2022 [3]. 

Despite significant progress in filovirus research, several questions remain unanswered or incompletely elucidated, such as the virus reservoir, transmission dynamics and spillover likelihood into human and animal populations, host range and susceptibility, and viral pathogenicity. Animal models, reverse genetics, and minigenome systems that are critical to developing specific countermeasures such as vaccines, diagnostic tools, and therapeutics are lacking for some filoviruses of public health importance. In addition, several newly emerging but poorly characterized filoviruses have been identified in bats during the last 15 years (Lloviu virus (LLOV), Bombali virus (BOMV), Měnglà virus (MLAV), and Dehong virus (DEHV)) [6,7,8,9]. In vitro studies suggest that these viruses could infect other mammals and potentially cause outbreaks in humans [6,7,8,9,10]. In this review, we summarize the current knowledge about the non-Ebola filoviruses BOMV, BDBV, RESTV, SUDV, TAFV, MARV, RAVV, LLOV, MLAV, and DEHV. Strategies for accelerating the development of effective countermeasures are also discussed. 

## 2. Orthoebolaviruses

### 2.1. Bombali Virus

**Figure 1:**
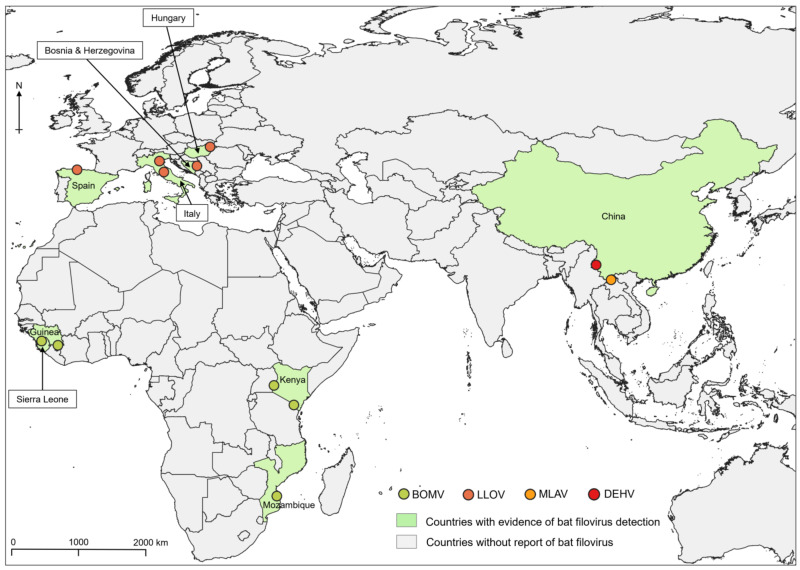
Locations of bat-derived filovirus detection. Countries in which bat-derived filoviruses have been detected by virus isolation or metagenomic analyses are shown in light green, with exact locations indicated by dots.

The complete genome of BOMV, representing the species *Orthoebolavirus bombaliense* [1], was first detected in free-tailed (*Chaerephon pumilus* and *Mops condylurus*) bats in Sierra Leone (Figure 1) using reverse transcriptase-polymerase chain reaction (RT-PCR) and next-generation sequencing in 2016 [7]. The virus was subsequently detected in Kenya, Guinea, and Mozambique in female Angolan free-tailed bats (*Mops condylurus*) (Table 1) [11,12,13,14]. It was found that cellular entry of recombinant vesicular stomatitis Indiana virus (rVSV) bearing BOMV GP was dependent on some host cell factors such as Niemann-Pick C1 (NPC1) that had been known to be essential for the cellular entry of other orthoebolaviruses and that the rVSV pseudotyped with BOMV GP could infect human embryonated kidney HEK293T and African green monkey kidney Vero cells [7].

Infectious BOMV has been generated by reverse genetics, and its pathogenicity has been evaluated in animal models [10,15]. IFNα/β receptor-knockout (IFNAR^−/−^) mice infected with BOMV survived with no signs of disease [15]. BOMV pathogenicity was low in HLA-A2-transgenic, NOD-scid-IL-2γ receptor-knockout mice reconstituted with human hematopoiesis, comparable to RESTV, which is believed to be apathogenic for humans [10]. Indeed, BOMV shares two specific amino acid residues with RESTV in its VP24 (positions 136 and 139), which are thought to be essential for the inability of RESTV to be pathogenic in humans [16]. These findings suggest that BOMV could be less pathogenic for humans. It was also shown that some previously generated monoclonal antibodies (mAbs) and convalescent plasma from EBOV survivors neutralized a lentivirus pseudotyped with BOMV GP [17].

Infectious BOMV has not yet been naturally isolated, and human infection has not yet been reported (Table S1). Therefore, it is important to conduct large-scale seroepidemiological surveys in human populations that have potential contact with the bat species mentioned above to assess the extent of exposure to BOMV. Finally, available reverse genetics and minigenome systems can be used to further study the biological properties of the virus and develop effective countermeasures (vaccines, therapeutics, diagnostic tools, etc.) to control or mitigate potential future spillover events. In fact, using a reverse genetics-based minigenome system for BOMV, it has been shown that remdesivir is active in inhibiting BOMV polymerase with IC_50_ values comparable to those against EBOV [18].

**Table 1:** Summary of non-Ebola filovirus outbreaks or primary cases.

Viruses	Year of Detection	Country	Human Cases	Deaths	CFR (%)
Filoviruses reported in humans [3,5,19,20] ^1^					
BDBV	2007	Uganda	131	42	32.1%
	2012	Democratic Republic of the Congo	38	13	34.2%
SUDV	1976	South Sudan	284	151	53.2%
	1979	South Sudan	34	22	64.7%
	2000–2001	Uganda	425	224	52.7%
	2004	South Sudan	17	7	41.2%
	2011	Uganda	1	1	100.0%
	2012	Uganda	17	7	41.2%
	2022	Uganda	164	55	33.5%
TAFV	1994	Ivory Coast	1	0	0.0%
MARV	1967	Ex. Yugoslavia	2	0	100.0%
	1967	Germany	31	7	22.5%
	1975	Zimbabwe	3	1	33.3%
	1980	Kenya	2	1	50.0%
	1990	Russia	1	1	100.0%
	1998–2000	Democratic Republic of the Congo	152	127	83.5%
	2004–2005	Angola	252	227	90.1%
	2007	Uganda	4	1	25.0%
	2008	USA	1	0	0.0%
	2008	Netherlands	1	1	100.0%
	2012	Uganda	15	4	26.6%
	2014	Uganda	1	1	100.0%
	2017	Uganda	4	3	75.0%
	2021	Guinea	1	1	100.0%
	2022	Ghana	3	2	66.6%
	2023	Equatorial Guinea	16	12	75.0%
	2023	Tanzania	8	5	62.5%
RAVV	1987	Kenya	1	1	100.0%
	1998–2000	Democratic Republic of the Congo	2	1	50.0%
Filoviruses reported in nonhuman hosts [7,8,9,11,12,14,21,22,23,24,25,26,27,28,29,30]					
BOMV	2016	Sierra Leone	- ^2^	-	-
	2018, 2019	Kenya	-	-	-
	2018, 2019	Guinea	-	-	-
	2015	Mozambique	-	-	-
RESTV	1989	USA	4 ^3^	0	0.0%
	1989	Philippines	3 ^3^	0	0.0%
	1992	Italy	-	-	-
	1996	USA	-	-	-
	1996	Philippines	-	-	-
	2008–2009	Philippines	6 ^3^	0	0.0%
	2015	Philippines	-	-	-
LLOV	2011	Spain	-	-	-
	2016–2020	Hungary	-	-	-
	2018–2021	Italy	-	-	-
	2022	Bosnia and Herzegovina	-	-	-
MLAV	2019	China	-	-	-
DEHV	2022	China	-	-	-

BDBV: Bundibugyo virus, BOMV: Bombali virus, RESTV: Reston virus, SUDV: Sudan virus, TAFV: Taï Forest virus, MARV: Marburg virus, RAVV: Ravn virus, LLOV: Lloviu virus, MLAV: Měnglà virus, DEHV: Dehong virus, CFR: case fatality rates, USA: the United States of America. ^1^ Confirmed cases. ^2^ There were no reported human cases. ^3^ Serological evidence. Workers developed antibodies but never experienced symptoms of the Ebola virus disease.

### 2.2. Bundibugyo Virus

**Figure 2:**
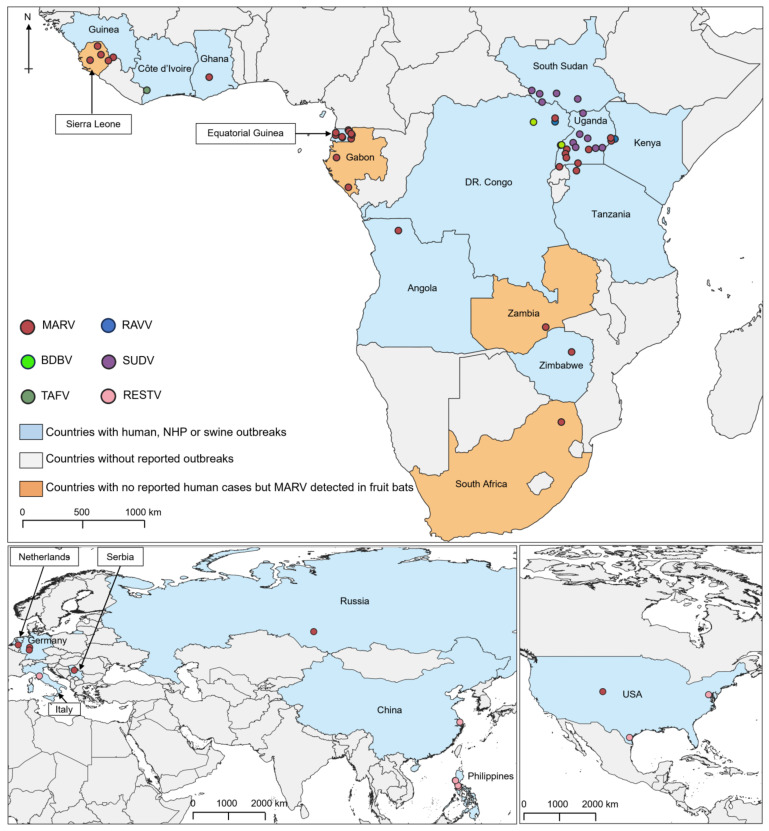
Locations of Ebola and Marburg disease outbreaks and primary cases caused by non-Ebola filoviruses in humans, NHPs, and pigs. Countries in which non-Ebola filovirus human or NHP outbreaks/primary cases, or swine cases were reported are shown in light blue. Countries in which MARV was detected in fruit bats but no human case has been reported are shown in pastel orange. Exact locations are indicated by dots. Outbreaks due to laboratory exposure (Russia) and historically reported outbreak epicenters in Germany and Serbia (ex-Yugoslavia) are depicted. Imported MARV cases in the United States and the Netherlands are also represented.

BDBV, currently the only virus representing the species *Orthoebolavirus bundibugyoense* [1], has been associated with two human viral hemorrhagic fever outbreaks clinically indistinguishable from EBOV, SUDV, or MARV infections (Table 1) [4,31]. The first outbreak, which led to the virus discovery, was recorded in August 2007 in two townships (Bundibugyo and Kikyo) of the Bundibugyo district in Uganda (Figure 2) [4,31]. However, the etiology of the hemorrhagic fever cases that appeared to be MARV or SUDV infections was initially unknown. It was later found that the virus genome was 30% different from the other known orthoebolaviruses, and this specific feature resulted in failures to confirm the first cases using RT-PCR assays available at the time and inaccuracies in reporting [31]. Eventually, the nearly complete viral RNA sequence was obtained using an isolate recovered in Vero E6 cells, and a BDBV-specific assay was developed [32]. The outbreak lasted nearly five months and affected between 116 and 149 persons, of whom 37–42 died [4,32,33,34]. In August 2012, BDBV reemerged with a severe hemorrhagic fever outbreak in Isiro (Figure 2), a city of the Haut-Uele province in the northeastern Democratic Republic of the Congo (DR. Congo), nearly 400 km from the Bundibugyo district [4,31]. Four complete BDBV genome sequences with 98.7% nucleotide and 98.9% amino acid identities with the Ugandan BDBV isolate were obtained from clinical samples [31,35]. The outbreak lasted three months with a total of 62 cases (36 laboratory-confirmed, 21 probable, and 5 suspected cases), including 34 deaths (with a case fatality rate of 54.8%) [35,36]. Alternatively, a case fatality rate of 34% has also been reported, considering other data (38 laboratory-confirmed cases, including 13 deaths) [31]. Sequence analyses of clinical samples from BDBV outbreaks in Uganda and the DRC showed a high degree of sequence identity among samples in each country, suggesting a single introduction, respectively, into the human population with subsequent human-to-human transmission [35]. However, a recent analysis of 11 additional BDBV genomes has challenged this previous hypothesis [31]. Of particular note, a defined pattern of progressive gain and loss of mutations to support intermediary genomic information from infected humans was lacking in the single spillover scenario [31]. It is currently believed that BDBV might have been introduced from unknown natural reservoirs into the human population via more than one spillover event during the 2012 outbreak [31]. To date, BDBV has not been shown to be associated with bats or NHPs, as neither the virus nor its genome has been detected in these animals (Table S1). Serological evidence (ELISA and Western blotting) of potential exposure to a virus antigenically similar to BDBV has been reported in 9/353 (2.6%) orangutans (*Pongo pygmaeus*) in Indonesia and 6/243 (2.5%) baboons (*Papio cynocephalus*)/vervet monkeys (*Chlorocebus pygerythrus*) and 8/748 (1.1%) fruit bats (*Eidolon helvum*) in Zambia, suggesting wide viral distribution beyond Eastern Africa [37,38,39]. 

Although BDBV infection clinically presents like EBOV, SUDV, and MARV infections [33,36,40,41,42,43], some differences were noted on the molecular level. BDBV infection is associated with low proinflammatory cytokine concentrations (e.g., IL-1α, IL-1β, IL-6, and TNF-α) and high anti-inflammatory cytokine concentrations (e.g., IL-10), which is distinct from EBOV infection [44]. The relatively small number of BDBV outbreaks has limited further pathological, immunological, and clinical characterization. In this context, animal models can offer better insights into these unexplored areas. Among the available animal models for BDBV infection, ferrets had 100% fatality within 8–9 days [45,46]. In a well-established model for EBOV infection, NHPs, especially cynomolgus macaques, had 50–75% lethality after BDBV challenge within nearly two weeks [45]. In IFNAR-/- mice, the virus was only lethal in about 3 mice out of 10 or adapted without inducing clinical disease or weight loss [4,45]. 

Several candidate vaccines have been developed and shown to be effective in preventing death after viral challenge in animal models. These candidates are either based on previously established viral vector vaccines (i.e., adenovirus and rVSV) [47,48] conferring cross-protection, chimeric antigens consisting of orthoebolavirus proteins [49,50,51,52], or BDBV-specific antigens [53]. Some of them could be positioned for clinical trials, building on lessons learned from recent advances in EBOV countermeasures. Another recently developed countermeasure is a BDBV minigenome system [54] that can be used in low containment facilities and has applications such as identification of antivirals through compound screening and elucidation of biological properties. The clinical management of BDBV infection solely relies on supportive measures, as there are no approved therapeutics. However, several human- and animal-derived broadly cross-reactive [55,56,57,58,59,60,61,62] or BDBV-specific mAbs [63] are available and could advance into clinical trials. The available diagnostic tools, in addition to classical viral isolation, sequencing, and electron microscopy, are now pan-filovirus RT-PCR [64,65,66] and, in some cases, rapid point-of-care tests detecting multiple species of orthoebolaviruses [67,68]. 

### 2.3. Reston Virus

RESTV, representing the species *Orthoebolavirus restonense* [1], was first isolated in 1989 during a viral hemorrhagic fever epizootic among captive cynomolgus macaques imported from the Philippines (Figure 2) and housed in a primate quarantine facility in Reston, Virginia [27]. Additional epizootics were reported in 1992 in Siena, Italy, and in 1996 in Alice, Texas, among cynomolgus macaques from the same Philippines exporting facility implicated in the initial 1989 epizootic (Figure 2) [28]. In 1996, another epizootic involving purpose-bred cynomolgus macaques occurred in the Philippines (Figure 2) [29]. Of a total of 458 occupationally exposed persons related to the monkey export facilities between 1989 and 1996, only five had serological evidence of exposure to RESTV [30]. RESTV reemerged nearly a decade later with an unexpected host. The virus was recurrently isolated from captive domestic pigs (*Sus scrofa*) in the Philippines in 2008–2009 and 2015 during the surveillance of porcine reproductive and respiratory syndrome (Table 1 and Table S1) [21,29]. Six of the 102 pig farm and slaughterhouse workers on the two affected farms tested positive using the CDC ELISA protocol [30]. All positive persons had daily occupational exposure to pigs, and none of them recalled any contact with bats or monkeys, suggesting the possibility of pig-to-human transmission [30]. Interestingly, RESTV RNA was detected in bats in the Philippines in 2010, further expanding the host range [69]. Beyond the Philippines, RESTV RNA has been detected in pigs in China [70], and serological evidence of exposure of pigs to RESTV has been reported in Uganda [71]. Similarly, antibodies reacting to RESTV antigens have been detected in NHP and bat samples from Zambia [37,38,39], Bangladesh [72], China [73], the Philippines [69,74], and Australia [75], and in orangutans from Indonesia [76]. However, infectious RESTV could not be isolated from the above samples, nor could the complete genome be assembled [4]. 

Little is known about the typical clinical features of RESTV infection due to coinfection with either simian arteriviruses in cynomolgus macaque epizootics or porcine arteriviruses, porcine reproductive and respiratory syndrome virus, and circoviruses in pig epizootics [21,77]. These coinfections make it difficult to tell whether the manifested symptoms are due to a RESTV infection. Available data on clinical presentation rely on a few results from experimental laboratory infections, demonstrating that RESTV replication in pigs is subclinical, with high viral replication levels in lung and lymphoid tissues from which the virus was first isolated in 1989 [77,78]. In addition, although asymptomatic, these pigs were able to shed the virus through the nasopharynx, strongly suggesting their roles as reservoir hosts of RESTV [77,78]. In cynomolgus macaques, it is suggested that RESTV infection is clinically and pathologically similar to EBOV and SUDV infections but presents with a slower progression and a lower lethality rate of 80–100% [4]. Of note, infection of humanized mice (hu-NSG-SGM3) with EBOV and RESTV tended to correlate with the difference in the disease pathogenesis of human infection, with EBOV replication being significantly higher in the liver than the spleen, unlike RESTV [79]. More recently, RESTV infection was found to be uniformly lethal in ferrets [80], making them a suitable animal model for evaluating pathogenicity and countermeasures when the typical highly lethal NHP model is logistically out of reach. It still remains elusive whether RESTV causes disease in humans, despite evidence of asymptomatic exposure to the virus [30]. The possibility that RESTV will adapt to humans through mutations under selective pressures and eventually pose a significant threat to human health cannot be strictly ruled out since some RESTV-susceptible hosts, such as pigs, are in the human food chain. Moreover, domestic pigs are now known to be susceptible to NHP-derived RESTV without requiring host species-specific viral adaptations (Table S1) [81].

Regarding countermeasure development, there is currently no vaccine available for RESTV because this virus is not considered pathogenic to humans. Minigenome and reverse genetics systems that can be used to characterize the biological properties and pathogenicity of RESTV are available [82,83]. Although information on RESTV-specific mAbs is lacking, some human-, mouse-, and monkey-derived broadly cross-reactive mAbs have been shown to neutralize pseudotyped VSV and/or authentic RESTV infection in vitro [59,60,84,85,86,87,88]. During the initial outbreak, RESTV infection was diagnosed using classical virus isolation, serology, and electron microscopy. Since then, several serological and nucleic acid-based assays to detect RESTV have been developed and can readily be used [65,76,89,90,91,92,93]. Field- and user-friendly RESTV-specific lateral immunochromatographic assays have yet to be developed, although some existing assays primarily targeting EBOV can detect RESTV as well [94].

### 2.4. Sudan Virus

SUDV, the second leading cause of Ebola disease, was discovered in Sudan in 1976 when it caused a Marburg disease-like outbreak [20,95]. The outbreak affected four towns (Nzara, Maridi, Tambura, and Juba) (Figure 2), with a combined community and nosocomial spread (Table 1) [95]. Two viral strains were then isolated from the acute-phase sera of two patients, and the diagnosis was additionally confirmed by immunofluorescence, pathology, and electron microscopy (Table S1) [95]. During the same year, the first-ever Ebola disease outbreak due to EBOV was recorded in DR. Congo [95]. Six more outbreaks caused by SUDV have been sporadically reported since 1976 in Sudan (1979 and 2004) and Uganda (2000–2001, 2011, 2012, and 2022) (Figure 2) [20,35,96,97,98], bringing the overall reported case number to 942, with 467 deaths (case fatality rate 49.6%) [20]. Nearly half of the cases were recorded during a single outbreak in the Gulu district of Uganda in 2000 (425 confirmed cases, with 224 deaths) [97]. Despite this high case load and recurrent outbreaks since 2000, SUDV has received less attention than EBOV in terms of countermeasure development. 

Several animal models exist for SUDV, including NHPs (50–100% lethality) [45], ferrets (100% lethality) [46], and IFNα/β receptor-knockout mice (5–100% lethality) (Table S1) [4,45]. Mice and ferrets could be useful for preliminary screening in resource-constrained settings before scaling up to logistically demanding NHP models. Approved therapeutic mAbs for Ebola disease (Inmazeb^®^ and Ebanga^®^) [99], which target specific epitopes of EBOV-GP, may not be active against SUDV. However, several promising pan-filovirus broadly neutralizing antibodies are either under development [55,56,57,58,59,60,61,62,100] or ready to progress to clinical trials, such as MBP134, which has been shown to prevent death in NHP models [101] (Table S1). Candidate therapeutic drugs such as remdesivir, a nucleotide analogue that inhibits viral RNA polymerase activity, have shown protective effects against lethal EBOV and other filoviruses in NHPs [102]. The use of remdesivir for SUDV is yet to be assessed, either in monotherapy or in combination with mAbs [103]. The combination therapy achieved higher (80%) protection in NHPs after the SUDV challenge than remdesivir or a mAb cocktail (MBP431) alone (20%) [104]. Three vaccine candidates were recommended by the World Health Organization for trials during the Uganda 2022 outbreak: rVSV-SUDV, ChAd3-SUDV, and ChAdOx1-biEBOV [105,106,107]. rVSV-SUDV is a monovalent chimeric rVSV carrying the SUDV GP gene, preventing lethal disease by SUDV in NHPs [108]. ChAd3-SUDV is a monovalent simian adenovirus vector vaccine encoding SUDV-GP and is already under a phase 1 open-label, dose-escalation clinical trial in Uganda [107], and ChAdOx1-biEBOV is a bivalent replication-deficient simian adenovirus vector vaccine, encoding EBOV and SUDV GPs [105]. Regarding diagnosis, although virus isolation, electron microscopy, high-throughput sequencing [109], pan-filoviruses RT-PCR [64,65,66], and lateral flow immunoassays [67,94] are available, they appear to be either not specific to SUDV or not readily available in resource-limited settings where they are most needed (Table S1). Available point-of-care testing kits primarily target EBOV, and some may additionally detect SUDV and other orthoebolaviruses without clearly discriminating among the respective infections, which are clinically indistinguishable [67,94]. SUDV research and development is hampered by the sporadicity of outbreaks and insufficient investment. Prioritizing SUDV countermeasure development, as recently seen during the Uganda 2022 outbreak, is critical for preventing future outbreaks and disease spreading beyond areas previously thought to be endemic. 

### 2.5. Tai Forest Virus

TAFV, the only virus of the species *Orthoebolavirus taiense* [1], is one of the lesser-known orthoebolaviruses. TAFV was found in 1994 when a Swiss ethologist got infected with this virus through a necropsy of a wild chimpanzee (Pan troglodytes verus) at the Parc National de Taï of Côte d’Ivoire (Figure 2) [110,111]. The chimpanzee originated from a troop that experienced a population decrease due to several epizootic hemorrhagic fever outbreaks in 1992 and 1994 [111,112]. Eight days after performing the necropsy, the ethologist presented with a febrile illness with high fever, headache, chills, myalgia, cough, abdominal pain, diarrhea, vomiting, weight loss, and a macular rash [110,111]. She eventually recovered six weeks later, without sequelae [110]. TAFV infection was confirmed by electron microscopy, serology, and virus isolation [110,113]. In addition, histopathological analyses of tissues from the necropsied ape showed lesions mimicking those observed in macaques experimentally infected with EBOV, strongly indicating human infection from the necropsied ape [4]. The origin of the chimpanzee’s infection is still unclear. Although antibodies reacting to the TAFV GP antigen have been detected in Indonesian orangutans, Ghanaian pigs, and Zambian fruit bats and NHPs [37,38,39,114], the virus has never been detected in any animals other than the initial and only human case (Table S1). This has limited further epidemiological, clinical, molecular, and immunological characterization of human infection. Furthermore, none of the 74 identified contacts of the initial patient tested positive, and human pathological data are lacking as biopsies could not be performed for the lone survivor [4,110]. However, zoonotic transmission is very plausible, and the possibility of future spillover events cannot be ruled out. Human infection with TAFV has been experimentally replicated only in cynomolgus macaques (*Macaca fascicularis* or Cynomolgus macaque), in which a partially lethal infection is observed [115,116]. Logistic and ethical constraints limit the use of NHP models, therefore undermining the development of countermeasures. Meanwhile, humanized mice, except for NSG-huPBL, can be used as small animal models, as a fatality rate of 18% has been reported 11-13 days after challenge with TAFV [45]. To date, there are no available minigenomes or reverse genetic systems for TAFV. 

Regarding vaccine development, rVSV expressing TAFV GP is the most promising as it has proven protective efficacy in an NHP model after lethal TAFV challenge [115], and this vaccine platform has been approved by the FDA for an EBOV vaccine. TAFV-specific mAbs are not available; however, potent cross-neutralizing [56,59,117,118] and cross-reactive [119] mAbs have been described (Table S1). TAFV infection can be diagnosed by real-time RT-PCR and genetic sequencing [65]. However, these testing modalities are not readily available, especially in resource-limited countries. Some lateral flow immunochromatographic assays can be useful and are field-friendly, although most of them cannot discriminate among orthoebolavirus species [68,94,120].

## 3. Orthomarburgviruses

### Marburg Virus and Ravn Virus

MARV and RAVV are two distinct viruses of the same species, *Orthomarburgvirus marburgense* [1]. Both viruses cause Marburg disease, which is a severe and often fatal hemorrhagic fever syndrome affecting both humans and NHPs, with a case-fatality rate as high as 90% [121,122,123]. In humans, the disease is characterized by non-specific symptoms such as fever, chills, general malaise, diarrhea, vomiting, odynophagia, and myalgia associated with a characteristic maculopapular rash and subtle or clear hemorrhagic symptoms [123]. The incubation period spans between 3 and 21 days, and death occurs within one to two weeks after the onset of symptoms [123]. MARV was concurrently discovered in Marburg and Frankfurt, Germany, and Belgrade, in the former Yugoslavia, in 1967 and has caused most of the 17 recorded Marburg disease outbreaks (Figure 2) [124,125]. On the other hand, RAVV was isolated from a Danish boy during routine clinical viral hemorrhagic fever surveillance in 1987 in Kenya and has been the causative agent of the other Marburg disease outbreaks clinically indistinguishable from MARV infection in DR. Congo and Uganda (Table 1) [124,126]. While MARV and RAVV are nearly 20% divergent, MARV has some variants with fewer genomic variations, such as Musoke, Durba, Angola, Ozolin, Ci67, Ratayczak, Voege, and Popp [127]. 

Most Marburg disease outbreaks occurred or originated in Africa, except outbreaks due to laboratory exposure in Russia in 1988, 1990, 1991, and 1995 (Figure 2) [121,124,128,129]. Of note, two MARV imported cases were reported in 2008 in the United States (Colorado) and the Netherlands (Helmond) (Figure 2) [130,131]. Both cases were female tourists who visited the Python cave in Queen Elizabeth National Park in Uganda and developed an unexplained febrile illness, which was later confirmed as MARV infection by serology or virus detection [130,131]. For many years, MARV was believed to be less threatening than EBOV. However, MARV regained attention when it caused two of the most significant outbreaks ever recorded, in the northeastern DR. Congo in 1998–2000 (154 cases, including one RAVV case, with 128 deaths) and northern Angola in 2004–2005 (252 cases with 227 deaths) (Figure 2) [132,133,134]. The DR. Congo 1998–2000 outbreak was particularly interesting as at least nine different MARV variants, including RAVV, were circulating among the tested patients, suggesting several distinctive spillovers from the natural reservoir to humans in a single outbreak [132,133,134]. In addition, 53 of the 154 cases were miners, and most of them (50/53) were linked to illegal underground gold mining activities in the Goroumbwa mine, suggesting the presence of reservoir hosts inside the mine and potential zoonotic transmission [4,134]. On the other hand, analysis of sequences from the Angola 2004–2005 outbreak indicated a single introduction of MARV [132,133,134]. To date, MARV has emerged in more countries and areas in Africa, as exemplified by the recent outbreaks in Guinea in 2021, Ghana in 2022, Equatorial Guinea in 2023, and Tanzania in 2023, bringing the overall caseload to 498 with 396 documented deaths, including three human infections recorded for RAVV, of which two were lethal [4,5].

MARV and RAVV are likely maintained by cave-dwelling fruit bats (*Rousettus aegyptiacus*), and humans are believed to be infected through contact with excretions or secretions of infected bats [135,136,137,138,139]. It is worth noting that MARV was found in this bat species even in countries (e.g., Gabon, South Africa, Zambia, and Sierra Leone) where the initial human cases have never been reported [135,136,138,140]. Humans may also be infected through contact with infected spillover hosts, such as NHPs, as observed during the first Marburg disease outbreaks in 1967 [125]. After that, human-to-human transmission will be triggered via exposure to the blood or bodily fluids (e.g., tears, semen, saliva, sweat, stool, urine, and breast milk) of an infected individual [129]. Exposure events such as administering medical care or handling infected dead bodies greatly amplify infection in health facilities and communities [129,141]. 

Marburg disease clinical diagnosis is often missed in the early phases due to similarities in clinical presentations with other febrile illnesses prevalent in Africa. Therefore, rapid and accurate laboratory diagnosis is critical. Several testing modalities are available or under development depending on the course of infection, including nucleic acid amplification tests (RT-PCR, qRT-PCR, RT-LAMP, etc.) and sequencing, antigen and antibody immunoassays (ELISA, lateral flow immunochromatographic test), and virus isolation/immunohistochemistry (Table S1) [123,142,143]. Diagnostic sample types are blood, bodily fluids, and tissue from an autopsy [123]. Virus isolation and immunohistochemistry require high containment facilities, highly skilled and trained human resources, and 7 to 10 days to complete [142]. Nucleic acid amplification tests, including hemorrhagic fever multiplex PCR, can be performed in reference laboratories and are available as in-house prototypes or commercial products, with results available within 3 h [142]. In-house MARV ELISA assays can yield results within 3–4 h [142]. A MARV NP-detecting lateral flow immunochromatographic test has been developed and is under evaluation [143], but the clinical validation of such promising tools that are capable of delivering diagnostic within 30 min could be challenged by the limited availability of well-characterized clinical samples.

Animal models commonly used for MARV infection include rodents (mouse, hamster, and guinea pig) and NHPs (cymomolgus macaque (*Macaca fascicularis*) and rhesus macaque (*Macaca mulatta*)) (Table S1) [144]. Cynomolgus and rhesus macaques best recapitulate human disease and pathology after challenge with wild-type MARVs, but their use is logistically and financially challenging [46,144]. Rodent models usually require a rodent-adapted MARV to cause the disease and mimic human infection [46,144,145,146,147]. Although ferrets are established as small animal models for orthoebolaviruses, infection with MARV or RAVV does not lead to any disease signs [46,144].

Reverse genetics and minigenome systems are available for MARV [148,149,150,151]. They are expected to contribute to accelerating MARV countermeasure development. Currently, there are no approved vaccines or therapeutics for Marburg disease worldwide, and approved EBOV vaccines do not offer cross-protection. However, the recent emergence of MARV in Guinea, Ghana, Equatorial Guinea, and Tanzania has underscored the critical need for effective vaccines to prevent future outbreaks [152]. As a result, the WHO R&D blueprint has set up a consortium for developing MARV vaccines through international collaboration (MARVAC) [152]. Several vaccines have been proven to have protective efficacy against MARV in preclinical NHP studies, with survival rates varying between 20 and 100% after lethal challenge with MARV [152]. These vaccines include whole virus, subunit (virus-like particle or GP), DNA, recombinant adenovirus, DNA/adenovirus, modified vaccinia Ankara vector (MVA)-BN-Filo, and rVSV vaccines [123,152]. Among MARV candidate vaccines, three are in phase I clinical trials: ChAd3-MARV, MARV DNA, and MVA-BN-Filo [123]. MVA-BN-Filo is scheduled for a phase 2/3 clinical trial [123]. The development of MARV therapeutics is also lagging as opposed to EBOV, for which clinical trials have resulted in the FDA approval of two drugs, Inmazeb^®^ and Ebanga^®^ [99]. Available MARV therapeutic approaches include small-molecule compounds (ribavirin, galidesivir, favipiravir, and remdesivir), interferon-beta, small interfering RNA, phosphorodiamidate morpholino oligomers with positive charges, MARV-specific mAbs (MR186, MR191, etc.), polyclonal concentrated IgG, and combinations of these approaches [123,152]. These therapeutics have shown protective efficacy in NHP models and could serve as a basis for human use [152]. Other MARV-specific or panfilovirus antibodies derived from human survivors or mice are available and could advance to clinical trials [153,154,155,156,157]. In the meantime, treatment during Marburg disease outbreaks consists mainly of supportive care, including the administration of fluids, antimicrobials, and blood transfusions [129].

## 4. Other Filoviruses Derived from Bats

### 4.1. Cuevavirus

#### Lloviu Virus

LLOV, a bat-derived filovirus classified into the *Cuevavirus* genus and representing the species *Orthoebolavirus lloviuense* [1], was discovered by RT-PCR techniques and high-throughput sequencing of internal organs collected from insectivorous bat (*Miniopterus schreibersii*) carcasses found in Cueva del Lloviu, Asturias, Spain, in 2011 (Figure 1) [22]. Infectious LLOV has been isolated from fresh Schreiber’s bat carcasses and blood collected in Hungary during the period of 2016–2020, as well as blood clot samples collected in northern and central Italy between 2018 and 2021 (Table 1) [23,24,25]. The isolated virus from Hungary could infect human and monkey cell lines, suggesting spillover potential [23]. In addition, LLOV has been detected in lung samples of dead Schreiber’s bats in Bosnia and Herzegovina [26]. All together, these findings support the role of Schreiber’s bats as natural hosts for LLOV in European countries. 

Studies using a replication-incompetent rVSV pseudotyped with the LLOV GP have shown that the LLOV GP plays a major role in viral tropism and pathogenicity and mediates viral entry through the NPC1 endosomal receptor in a pH- and cathepsin L-dependent manner [4]. LLOV GP uses human C-type lectins for attachment to cell surfaces and enters a cell similarly to other known orthoebolaviruses and orthomarburgviruses. In addition, LLOV GP has the potential to mediate infection of cell lines of different origins, including human, monkey, and pig, while showing preferential tropism for some bat cells [158,159]. Other studies have reported the establishment of LLOV minigenome systems based on the complementation of missing genome ends with sequences from other filoviruses [160,161]. These systems have revealed that the mechanisms of replication and transcription of LLOV are more similar to those of orthoebolaviruses than to those of orthomarburviruses [160,161]. The possibility of LLOV causing diseases in humans, NHPs, and other mammalian species is still unclear. However, the pathogenicity of LLOV has been assessed in type I interferon receptor knockout (IFNAR-/-) mice using a low and high dose of infectious LLOV via intranasal and intraperitoneal routes [15]. Increased viral loads were observed in the intraperitoneally infected high-dose LLOV groups after infection, but all the mice infected with LLOV survived with no sign of disease [15]. Further experiments using other animal models are needed to better elucidate the pathogenicity of LLOV.

Serological assays for the detection of specific antibodies to LLOV have been developed, including neutralization tests with pseudotyped viruses expressing LLOV GP [23,158], and a soluble purified GP-based enzyme-linked immunosorbent assay (ELISA) (Table S1) [158]. These assays showed that LLOV is serologically distinct from other known filoviruses. Serological evidence of exposure to LLOV has been confirmed in Schreiber’s bats in European countries [23,162], domestic pigs in Indonesia (unpublished data), and in one Egyptian fruit bat in Zambia [163]. The detection of specific IgG antibodies to LLOV in animals in Southeast Asia and Africa suggests its wide distribution and calls for further studies to understand the ecology of this filovirus outside of the European continent. Serological studies of human populations are necessary to shed light on the prevalence of this virus, given the presence of bats in the food chains of several countries.

Regarding countermeasure development, an in vitro study evaluated the efficacy of four antiviral drugs against recombinant LLOV using bat (SuBK12-08) and human (Huh7) cell lines in comparison with recombinant EBOV and found similar virus inhibition patterns in both cell types by remdesivir and U1866A (an inhibitor of the filovirus receptor Nieman-pick) [160]. While LLOV was more sensitive to universal type I IFN pretreatment than EBOV, mAb114 (Ebanga^®^), an approved therapeutic against EBOV, had no effect on LLOV [160], confirming that translation of the countermeasures proven to be effective against EBOV may fail for LLOV. Therefore, the development of LLOV-specific countermeasures, including vaccines and mAbs, is needed, given its potential to infect humans. Similarly, molecular, serological, ecological, and environmental studies of Schreiber’s bats should be undertaken to predict transmission routes and identify additional potential hosts.

### 4.2. Dianlovirus

#### Měnglà Virus

MLAV, a new filovirus reported in 2019 representing the species *Dianlovirus menglaense* [1], is phylogenetically distinct from the other members of the family *Filoviridae* and classified in the genus *Dianlovirus*. MLAV was detected in the liver of a fruit bat (*Roussetus* spp.) captured in Měnglà County, Yunnan Province, China (Figure 1) [8]. The complete MLAV genome shares 32–54% nucleotide sequence identity with other known mammalian filoviruses but is biologically related to the orthomarburgviruses [8]. Recently, filovirus RNA genomes were detected in *Roussetus* bats caught in Vietnam, and phylogenetic analysis of its polymerase L fragment revealed the presence of viruses belonging to the *Dianlovirus* genus [164]. This finding suggests that dianloviruses may exist not only in China but also in other parts of the world.

To date, infectious MLAV has not yet been isolated (Table S1). Studies with rVSV pseudotyped with MLAV GP revealed broad cell tropism, suggesting its potential for spillover transmission to other animals, including humans [8,165]. In addition, as observed for other mammalian filoviruses, MLAV GP was shown to depend on proteolytic digestion by host proteases such as cathepsin L and to use NPC1 as an endosomal receptor for viral entry into cells [8,165]. An in vitro study using a lentivirus-based pseudotype system revealed that some FDA-approved drugs (not for filovirus diseases) that inhibited EBOV and MARV entry (Paroxetine, Imipramine, Bepridil, Dibucaine, Orphenadrine, Benztropine, Fluoxetine, and Sertraline) also inhibited MLAV entry into cells. In addition, analysis of anti-MARV GP neutralizing mAbs (MR-78 and MR-191) and ADI-15878 known to display cross-reactivity to all known orthoebolaviruses showed that only MR-191 neutralized the MLAV GP-pseudotyped virus, but with a lower potency than MARV (Table S1) [165]. An MLAV minigenome system consisting of MLAV polymerase complex, leader, and trailer sequences that exhibit intrinsic specificities compared to those of MARV and EBOV has been recently developed [166]. It was also found that the MLAV minigenome transcription and replication activities were independent of VP30 [166]. Using that system, remdesivir was found to efficiently inhibit MLAV genome replication (Table S1) [166]. The MLAV minigenome system is expected to be critical for antiviral screening and to advance countermeasure development for this novel filovirus.

### 4.3. Currently Unclassified Bat-Derived Filovirus

#### Dehong Virus

DEHV, the most recently discovered filovirus, was isolated from Chinese fruit bats (*Roussetus leschenaultii*) after a decade of sustained surveillance activities on bat filoviruses in Dehong prefecture, Yunnan province, China (Figure 1) [9]. The isolated virus has a filamentous shape like other filoviruses [9]. Phylogenetic analyses suggested that DEHV could be classified in a new genus (putative *Delovirus*) between the genera *Dianlovirus* and *Orthomarburgvirus*, considering the low genome sequence identity, the largest genome size within filoviruses (20,943 nucleotides), and the nucleotide frequency pattern with the highest AU content among filoviruses (68.2% vs. 54.0–63.5%) [9]. Continuous detection of DEHV RNA and serological data has confirmed the circulation of this virus in bats in Yunnan province [9].

It has been shown that DEHV uses the NPC1 receptor for cellular entry like other filoviruses and is sensitive to remdesivir-induced replication inhibition [9]. DEHV displays broad cell tropism in vitro, but its pathogenicity to bats and other mammalian hosts is unclear. Regarding antigenicity, anti-DEHV NP sera intensely cross-react with MARV and MLAV NPs and weakly with LLOV NP [9]. The antigenic characterization of GP has not been reported. As for other emerging bat-derived filoviruses, DEHV spillover to humans and subsequent human-to-human transmission cannot be ruled out since the circulation of DEHV has been proven in bats that share a close interface with humans. However, like RESTV, DEHV is thought to be less pathogenic for humans than all known human-pathogenic filoviruses (Table S1) [9]. Experimental infection studies using animal models will be critical to gaining a better understanding of the pathogenicity and host range of DEHV. Similarly, the generation of minigenome and reverse genetics systems will be critical to supporting the development of public health countermeasures. Finally, large-scale serological surveys using available techniques such as ELISA, indirect immunofluorescence assays, and neutralization assays will be useful to assess exposure to DEHV in bat and human populations beyond the Yunnan region in China as well as to understand the ecology of this virus.

## 5. Discussion

Research on filoviruses has progressed since the discovery of MARV in 1967. This is particularly true for EBOV for which major advancements in outbreak preparedness and control, vaccines, therapeutics, and diagnostics have been made. Most milestones happened after the 2013–2016 West Africa outbreak, which enhanced global awareness of the need to urgently and jointly address the filovirus threat to achieve global health security. However, this global commitment to advance research and development exclusively on EBOV has been to some extent detrimental to research on equally devastating non-Ebola filoviruses causing human outbreaks more sporadically (MARV, RAVV, BDBV, TAFV, and SUDV). Indeed, MARV outbreaks have recently been reported during the last 3-year period in areas not previously affected [5]. Moreover, several poorly characterized bat-derived filoviruses emerged recently (LLOV, BOMV, MLAV, and DEHV) and have been suggested to have the potential to infect several hosts, including humans [6,7,8,9]. Some of these bat-associated filoviruses share similarities with known deadly filoviruses (e.g., EBOV and MARV) and could represent serious threats for both humans and animals. Several other non-Ebola filoviruses have been associated with symptomatic or asymptomatic human infections (BDBV, TAFV, and RESTV) and have drawn less attention, while their pathogenic potential for humans is not fully understood. 

Several challenges hamper the control of non-Ebola filoviruses, especially in resource-limited countries. These include difficulties in laboratory diagnosis, disease surveillance, and control, as well as research and development. Addressing these challenges is critical since infectious diseases can spread rapidly with international travel and trade. Deadly outbreaks starting in an endemic country can spread beyond borders and threaten global health security. Regarding laboratory diagnosis, although high-throughput sequencing methods are effective in detecting known and novel/unknown viruses, they are not readily available, especially in African countries where most epidemics break out. Most African countries lack proper infrastructure, trained human resources, sustainable supply chains, and logistics for reagents, resulting in poor testing capacities [124]. Field- and user-friendly point-of-care diagnostic tools targeting non-Ebola filoviruses are either inexistent or lack specificity. In that context, establishing or strengthening high-containment regional or subregional reference laboratories is crucial to mutualizing available resources and decreasing facility maintenance costs in individual countries. These laboratories would network effectively with existing national public health laboratories, working toward disease surveillance, outbreak preparedness, and response. Moreover, regional collaboration creates a platform for human resource capacity building, best practices, and experience sharing from countries with advanced expertise and experience in filovirus outbreak prevention, detection, and response. Disease surveillance and control can be strengthened through regional experience sharing from countries previously affected by filoviruses. Sharing best practices on case finding and isolation, contact identification and tracing, risk communication and community engagement, case management, and infection prevention and control can enhance preparedness against outbreaks caused by filoviruses in non-affected countries. 

Research and development on non-Ebola filoviruses is also challenged by issues such as limited public support, waning of funding during non-outbreak periods, unavailability of isolated viruses or surrogate systems (reverse genetics, minigenome, and pseudotype systems), lack of typical samples, and limited animal models for virus characterization and pathogenicity assessment. As exemplified during the COVID-19 pandemic, research and development funding gaps can be filled by promoting joint venture schemes with diverse shareholders (government, industry, academia, and non-profit and non-governmental organizations). These schemes have the potential to increase resource mobilization. A promising example is the WHO-coordinated MARV vaccine consortium, MARVAC, which is a platform for joint clinical trials and assay evaluation, mutualization of resources, and international research collaboration [152]. Such initiatives can be translated to other neglected non-Ebola filoviruses to advance research and the development of critically needed countermeasures. The unavailability of isolated viruses can be addressed by building local capacities in sample collection, storage, and transportation to designated regional or subregional reference laboratories. These laboratories’ capacities to conduct virus isolation or organize the shipment of dangerous pathogens to higher containment facilities can be strengthened through collaborative research. Collaborative research, especially during outbreaks, will be critical to establishing clinical sample biorepositories. The latter can be used for virus isolation, characterization, and the development or validation of countermeasures such as lateral flow immunochromatographic assays or therapeutics. Efforts to further develop and expand the use of low-containment surrogate systems should be undertaken in parallel to those for virus isolation. These systems have the potential to decrease BSL-4 dependency and advance basic research and countermeasure development. Lessons learned from the use of minigenomes, reverse genetics, and pseudotype systems for Ebola virus, influenza virus A, rabies virus, and, recently, SARS-CoV-2 could improve the current status of preparedness against non-Ebola filoviruses [167,168]. Regarding animal models, additional species capable of mimicking human infection and modeling the full disease spectrum are needed for assessing virus pathogenicity. Exploring alternatives such as organoids could address logistic constraints and ethical issues associated with animal use, especially NHPs. More studies comparing filovirus organoids and animal models will provide useful insights into these promising alternatives. Finally, strong political leadership and commitment are needed to promote multisectorality, multidisciplinarity, and international collaboration to accelerate filovirus research and enhance outbreak preparedness and response. Political commitment is also expected from regulatory agencies such as the WHO and FDA to grant exceptions to expedite approval for critically needed products such as diagnostic tests, vaccines, and drugs.

## Data Availability

Not applicable.

## Supplementary Materials

The following supporting information can be downloaded at: https://www.mdpi.com/article/10.3390/v16081179/s1, Table S1. Host range, pathogenicity, and available countermeasures for non-Ebola filoviruses.
