# Supplementary material for: Non-Ebola Filoviruses: Potential Threats to Global Health Security
Source: Viruses. 2024 Jul 23;16(8):1179. doi: 10.3390/v16081179 (PMC11359311; doi:10.3390/v16081179)
Supplement: Supplementary file 1 [file viruses-16-01179-s001.zip › viruses-3096354-supplementary.pdf]

Table S1. Host range, pathogenicity and available countermeasures for non-Ebola filoviruses

Viruses	Infected hosts[1]	Pathogenicity to humans	Virus isolation	Countermeasures						
				Minigenome	Reverse genetics	Animal models	Vaccine candidates	Small molecules	Potential therapeutic antibodies	Diagnostic tools
BOMV	Bats [7,11–14]	Unknown	No	Yes [18]	Yes [18]	HLA-A2-transgenic, NOD-scid-IL-2γ receptor-knockout mouse [10]	No	Yes [18]	Cross-neutralizing Ebola virus antibodies [17]	Sequencing, RT-PCR [7,11–14]
BDBV	Humans, NHPs, bats [4,31,37–39,163]	Yes [4,31]	Yes [32]	Yes [54]	No	Ferrets, NHPs, IFNAR-/-, humanized mouse [45,46]	Yes [47–53]	No	Broadly cross-reactive or BDBV-specific mAbs [55–63]	Sequencing, RT-PCR, ELISA, Westernblot, LFI [32,37–39,64–68]
RESTV	NHPs, pigs, bats, humans [21,27,71–75,163,28–30,37–39,69,70]	No[2]	Yes [21,27,29]	Yes [82,83]	Yes [82,83]	Pigs, NHPs, humanized mouse, ferrets [4,45,77–80]	No	No	Broadly cross-reactive mAbs [59,60,84–88]	Sequencing, RT-PCR, ELISA, LFI [65,76,89–94]
SUDV	Humans, NHPs, bats [20,35,37–39,95–98,163]	Yes [20,35,95–98]	Yes [95]	No	Yes [108]	NHPs, ferrets, IFNAR-/- mouse, humanized mouse [4,45,46]	Yes [105,106,108]	No	Broadly cross-reactive mAbs [55–62,100]	Sequencing, RT-PCR, LFI) [35,64–67]
TAFV	Humans, NHPs, bats, pigs [37–39,110–112,114,163]	Yes[3]	Yes [113]	No	No	NHPs, humanized mouse [45,115,116]	Yes [115]	No	Broadly cross-reactive mAbs [56,59,117–119]	Sequencing, RT-PCR, LFI [65,68,94]

MARV	Humans, NHPs, bats [121–123,125,135–138,140]	Yes [5,121,134,122–127,132,133]	Yes [125,137, 139,140]	Yes [148,151]	Yes [149,150]	Mouse, hamster, guinea pig, NHPs [46,144–147]	Yes [123,152]	Yes [123,152]	Yes [153–157]	Sequencing, RT-PCR, RT-LAMP, ELISA, LFI [123,142,143]
RAVV	Humans, bats [121–123,125,135–138,140]	Yes [5,124,126,132, 134]	Yes [126]	Yes [148,151]	Yes [149,150]	Mouse, hamster, guinea pig, NHPs [46,144–147]	Yes [123,152]	Yes [123,152]	Yes [153–157]	Sequencing, RT-PCR, RT-LAMP, ELISA, LFI [123,142,143]
LLOV	Bats [22–26]	Unknown	Yes [23–25]	Yes [160,161]	Yes [161]	IFNAR-/- mouse [15]	No	Yes [161]	No	Sequencing, RT-PCR, ELISA, neutralization tests [23,158,162,163]
MLAV	Bats [8]	Unknown	No	Yes [8,166]	No	No	No	Yes [166]	MARV-Cross-reactive mAb [165]	Sequencing, RT-PCR [8]
DEHV	Bats [9]	Unknown	Yes [9]	No	No	No	No	Yes [9]	No	Sequencing, ELISA, IFA, neutralization assays [9]

BDBV: Bundibugyo virus, BOMV: Bombali virus, RESTV: Reston virus, SUDV: Sudan virus, TAFV: Taï Forest virus, MARV: Marburg virus, RAVV: Ravn virus, LLOV: Lloviu virus, MLAV: Měnglà virus, DEHV: Dehong virus, ELISA: Enzyme linked immunosorbent assay, LFI: Lateral flow immunoassay, IFA: Immunofluorescence assay, RT-PCR: Real-time reverse transcriptase polymerase chain reaction, NHP: Nonhuman primate, IFNAR-/-: interferon (alpha and beta) receptor 1 knockout, mAb: monoclonal antibody.

[1]Confirmed infection and serological indication.

[2]Although no human diseases have been reported so far, RESTV is highly pathogenic to NHPs.

[3]Only one human case was reported.
